# Morphine versus methadone for neonatal opioid withdrawal syndrome: a randomized controlled pilot study

**DOI:** 10.1186/s12887-022-03401-3

**Published:** 2022-06-15

**Authors:** Mary Beth Sutter, Hannah Watson, Nicole Yonke, Sherry Weitzen, Lawrence Leeman

**Affiliations:** 1 Department of Family Medicine, Alpert Medical School of Brown University, Providence, RI USA; 2 Santa Rosa Community Health, Santa Rosa, CA USA; 3 Family Medicine of Southwest Washington, Vancouver, WA USA; 4 Baystate Health, Springfield, MA, USA; 5 Department of Family and Community Medicine, Department of Obstetrics, University of New Mexico, Albuquerque, NM USA

**Keywords:** Neonatal opioid withdrawal syndrome, Perinatal substance use disorders, Methadone

## Abstract

**Background:**

Neonatal Opioid Withdrawal Syndrome (NOWS) is a significant public health issue and while millions of neonates are affected each year, an optimal pharmacologic weaning protocol has yet to be demonstrated. In this study, we compare hospital length of stay (LOS) and length of treatment (LOT) for treatment of neonatal opioid withdrawal (NOWS) with morphine versus methadone.

**Methods:**

This was a single-site, open-label, randomized controlled pilot study conducted from October 2016-September 2018. Infants were eligible if their primary in-utero drug exposure was heroin, oral opioids, or methadone and they were born at greater than or equal to 34 weeks gestation. Infants were excluded for serious medical comorbidities and primary in-utero exposure to buprenorphine.

**Results:**

Sixty-one infants were enrolled; 30 were randomized to methadone treatment, and 31 to morphine treatment. Overall 46% of infants required treatment for NOWS. LOS and LOT for infants treated with morphine was 17.9 days and 14.7 days respectively, compared to 16.1 days and 12.8 days for babies treated with methadone (*p* = 0.5, *p* = 0.54). Infants treated with morphine received lower total morphine equivalents than those treated with methadone (9.7 vs. 33, *p* < 0.01). Three treated infants in the methadone group required transfer to the Neonatal Intensive Care Unit, versus no infants in the morphine group.

**Conclusions:**

Infants treated with morphine versus methadone had no significant differences in LOS or LOT in this pilot study. Infants treated with methadone received up to 3 times the opioid based on morphine equivalents as infants treated with morphine and had more transfers to the NICU for over sedation.

**Clinical trial registration:**

Morphine Versus Methadone for Opiate Exposed Infants With Neonatal Abstinence Syndrome NCT02851303, initiated 01/08/2016.

**Supplementary Information:**

The online version contains supplementary material available at 10.1186/s12887-022-03401-3.

## Introduction

Neonates exposed to opioids in utero can develop a constellation of withdrawal symptoms known as neonatal opioid withdrawal syndrome (NOWS) that includes CNS irritability, gastrointestinal side effects and autonomic dysfunction [1–3]. Infants with NOWS are at risk for medical complications including failure to thrive, seizures, and prolonged neonatal hospital stays [1–3]. A recent analysis of three state Medicaid programs demonstrated that infants born to mothers with an opioid use disorder had high rates of NICU admissions, prolonged hospital stays, and higher health care costs compared to infants of women without a substance use disorder [4]. From 2004 to 2016 there has been a greater than fivefold increase in the rate of NOWS, from 1.6 per 1,000 hospital births in 2004 to 8.8 per 1,000 hospital births in 2016 [5]. The average length of stay for infants with NOWS is 16.9 days, and admissions for NOWS account for an estimated $1.5 billion in hospital charges [6, 7].

Non-pharmacologic treatment including rooming-in and breastfeeding have been shown to decrease the severity of withdrawal and length of stay for infants with NOWS [8–10], however between 21 to 94% of exposed infants develop signs of withdrawal that are severe enough to warrant pharmacologic treatment [1, 11, 12]. Investigators have shown that standardized weaning protocols decrease neonatal opiate exposure and length of hospital stay [13, 14]. There are a wide variety of protocols for pharmacological treatment of NOWS, with morphine and methadone being the most common first line treatments [15–17] and buprenorphine under study as an alternative [18–20]. Evidence surrounding the optimal treatment regimen is conflicting. While one study comparing methadone to morphine for infants with NOWS demonstrated no significant difference in length of hospital stay [21], others have found a shorter length of stay with methadone [22–24]. The most recent Cochrane review showed that the type of opioid used to treat NOWS has no effect on treatment failure, but with only low certainty evidence available [25]. In general, studies use different weaning protocols, involve varied levels of inpatient care, and have not calculated morphine equivalents between study arms, making direct comparisons between morphine and methadone difficult. Our study aimed to examine hospital length of stay and length of treatment of infants with neonatal opioid withdrawal treated with morphine versus methadone. In particular, our study adds the perspective of a different patient population with a non-NICU model of care. Our hypothesis was that morphine would result in a shorter length of stay and lower total morphine equivalent use as compared to methadone based on our institutional observational data in years prior.

## Methods

This was a single-site, open-label, randomized controlled pilot study conducted from October 1, 2016 to September 30, 2018 of morphine versus methadone treatment for infants who developed neonatal opioid withdrawal syndrome and required pharmacologic treatment. This study is registered on ClinicalTrials.gov since 01/08/2016 under NCT02851303. The primary research endpoint was length of hospital stay. Secondary research endpoints included length of treatment, need for a second treatment agent, total morphine equivalents received during hospitalization, need for assisted nutritional or feeding support (including increased calorie requirement and speech therapy intervention or gavage feeds), breastfeeding, and adverse events. The protocol was reviewed and approved by the university Institutional Review Board.

Infants were eligible if they were born at greater than or equal to 34 weeks gestation, and their primary in-utero drug exposure was methadone, oral opioids, or heroin, as evidenced by maternal history and a maternal or infant urine drug screen positive for these opioids on admission. Infants were excluded if they had any serious medical comorbidities, and if the primary in-utero substance exposure was buprenorphine (standard of care at the study site was to treat these infants with a morphine protocol [26].) Initial exclusion criteria excluded any neonatal intensive care unit admission. In April 2017, revised inclusion criteria allowed neonatal intensive care unit admission of less than 24 h and expanded the enrollment period from the prenatal care period to 24 h after delivery.

Parents or guardians of eligible infants were approached to offer enrollment and randomization within 24 h of birth, and consent was obtained by written method. Randomization was performed at the time of consent to eliminate barriers to medical care if need for infant pharmacotherapy were to occur after hours. Concurrent substance use during pregnancy was assessed by urine toxicology screen at the time of birth, and tobacco use was assessed by verbal screening. The hospital urine toxicology screen does not include marijuana metabolites, and marijuana use is assumed to occur with commonality.

Infants were admitted to a WHO Baby-Friendly newborn service that encouraged rooming in and breastfeeding if eligible. Infants with increased medical needs or no parent for rooming in were admitted to a small family accessible Level 2 nursery with 24 h a day visiting. All infants exposed to opioids in utero underwent a 96-h observation period with Finnegan Neonatal abstinence scoring according to hospital protocol [27]. Infants who met criteria for pharmacologic management with two Finnegan scores of ≥ 12 or three sequential combined scores of greater than 24 initiated methadone or morphine treatment according to the existing hospital protocols based on prior randomization (Additional file 1: Methadone and Morphine Treatment Protocols). Monitoring for each group on the rooming in floor consisted of an oxygen saturation and respiratory rate 30–60 min after the first two doses of methadone or morphine, and 30–60 min after each dose increase. Oxygen saturation and respiratory rate were monitored further each shift and as needed. Infants in the level 2 nursery received continuous cardiorespiratory and oxygen saturation monitoring. Infants who did not require pharmacologic treatment were excluded from the final analysis. The clinical management during the remainder of the hospital course, including the decision to start clonidine as an adjunctive non-opioid agent, was left to the discretion of the on-call attending physician. After completing the pharmacologic wean, infants were observed per hospital protocol for at least 48 h for signs of recurrent withdrawal requiring reinstitution of pharmacological treatment. Total morphine equivalents given during the hospital stay was calculated using the conversion factor of 4:1 for methadone:morphine [28].

Randomization was computer generated by investigators into blocks of 10 with assignment to either methadone or morphine and was concealed to investigators prior to the start of the study. Immediately after each consent was obtained, the next concealed randomization envelope was opened to eliminate barriers to care at the time of need for infant pharmacotherapy. Data was analyzed using STATA 14.0. Continuous variables were summarized as means or medians. Differences in maternal and neonatal characteristics between methadone and morphine groups were compared using bivariate analyses. Length of stay was summarized as mean and median, and the length of stay was compared between the morphine and methadone group using parametric and non-parametric methods. A sample size calculation done prior to the start of this pilot study using unpublished retrospective observational institutional data from prior years predicted a sample size necessitating > 5 years of study. In the interest of contributing our institutional experience and attempting to modernize the approach to care, it was decided to pursue a pilot study with a sample size too small to power for anything but large differences between treatment groups.

## Results

**Fig. 1:**
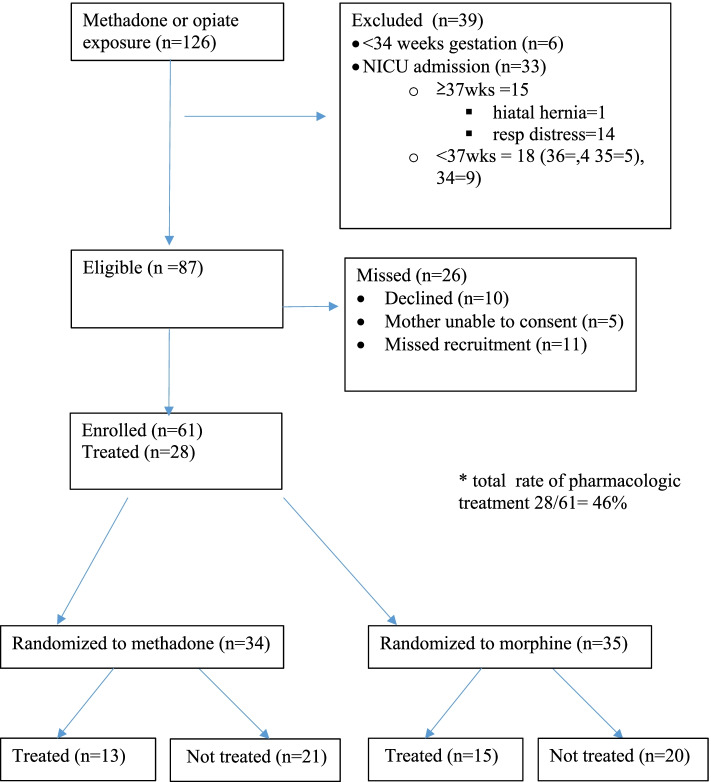
Screen, exclusion, consent rate, randomization, and treatment completion by study group (10/1/16- 9/30/18). NICU = Neonatal intensive care unit.The total rate of neonatal opioid withdrawal requiring pharmacologic treatment was n = 28 or 46%

During the study period, 126 methadone or heroin-exposed infants were admitted to the University of New Mexico Hospital with 61 neonates recruited into the study (Fig. 1). Thirty-nine infants were excluded from study and 26 of the eligible couplets were not recruited due to parents declined or were unable to give consent. The study ended in September of 2018 due to a desire to change the global hospital policy for treatment of NOWS, no safety stopping rules were applied. The baseline demographic characteristics between the morphine and methadone groups are displayed in Table 1. Mothers of infants in the methadone group were more likely to have initiated prenatal care in the first trimester (69% vs 33%), however the two groups had a similar number of prenatal visits. Other substance use was common in both groups; however, the morphine group had higher rates of tobacco use (46% vs 60%), methamphetamine (12% vs 66%), benzodiazepine (0% vs 26%), and SSRI use (0% vs 13%). Cocaine use was very low in both groups, and marijuana use was not assessed, as described in the Methods section.

**Table 1:** Maternal characteristics by study arm

Maternal characteristics	Methadone *N* = 13 Mean ± SD N(%)	Morphine N = 15 Mean ± SD N(%)
Age	25.4 ± 3.1	27.8 ± 4.2
Race/Ethnicity		
White non-Hispanic	6(46.1%)	2(13.3%)
Hispanic	7(53.8%)	11(73.3%)
Native American	0	2 (13.3%)
Initiation of prenatal care in 1^st^ trimester	9(69.2%)	5 (33.3%)
Number of prenatal visits	6.6 ± 4.4	4.8 ± 4.41
Concurrent illicit opioid use	10(76.9%)	12 (80%)
Concurrent tobacco use	6 (46.15%)	9 (60%)
Concurrent methamphetamine use	2 (12.38%)	10 (66.67%)
Concurrent benzodiazepine use	0	4(26.67%)
Concurrent alcohol use	0	0
Concurrent SSRI	0	2(13.3%)
Preterm delivery	2(15.4%)	4 (26.7%)
Maternal gestational diabetes	1(7.7%)	0
Maternal Preeclampsia	2(15.4%)	1(6.7%)
Gestational age at delivery	37.5 ± 1.2	37.7 ± 1.7
Birthweight (grams)	2721 ± 364	2981 ± 438
Vaginal delivery	11(84.62%)	13(86.7%)
Cesarean delivery	2(15.38%)	2(13.3%)

*SSRI* selective serotonin reuptake inhibitor

Length of hospital stay was not statistically different among the two treatment groups (methadone 16.1 versus morphine 17.9 days, *p* = 0.50) (Table 2). Despite a similar length of stay and length of treatment, infants in the methadone group received significantly more morphine equivalents of medication (33 vs. 9.68, *p* < 0.05). One neonate in the study that was randomized to morphine required clonidine. Notably, 3 treated infants in the methadone group required transfer to the neonatal intensive care unit due to over sedation as determined by a decreased respiratory rate and/or oxygen saturation. More infants in the morphine group required additional caloric support, but this difference was not statistically significant (73% vs 46%, *p* = 0.25). Infants in the methadone group spent more time rooming in, but this difference was also not statistically significant (52.9% of the hospital stay vs 30%, *p* = 0.18). Due to hospital policies, several infants were unable to room in due to involvement of child protective services, or absence of a parent during the hospital stay for other social or medical reasons.

**Table 2:** Neonatal outcomes by treatment arm

	Methadone Mean ± SD N(%)	Morphine Mean ± SD N(%)	*p*-value
Length of treatment (days)	12.8 ± 5.8	14.7 ± 9.9	0.54
Length of hospital stay (days)	16.1 ± 4.7	17.9 ± 8.6	0.50
Cumulative morphine equivalents (mg)	33.0 ± 17.49	9.68 ± 12.92	< 0.05
Need for additional agent (clonidine)	0	1 (6.7%)	1.0
NICU transfer	3 (32.1%)	0	0.09
Need for increasing kcal nutrition	6(46.1%)	11(73.3%)	0.25
Time spent rooming in (% of hospital stay)	52.9%	30%	0.18
Breastfeeding at discharge	5(38.5%)	5(33.3%)	0.78

*NICU* neonatal intensive care unit, *kcal* kilocalories

## Discussion

This study showed no significant difference between length of stay or length of treatment between infants treated with methadone or morphine for neonatal opioid withdrawal syndrome due to in-utero heroin, methadone, or illicit opioid exposure. There was a trend towards a shorter length of stay and length of treatment for the methadone group, which may have showed statistical difference with a higher sample size, as was found in other studies [21–23]. This trend may be explained by a higher rate of rooming in for infants in the methadone treatment group, which is known in the literature to decrease withdrawal symptoms and need for treatment [6–8]. Use of methadone, because it is long acting, requires less nursing interaction for medication administration and therefore may be more compatible with a rooming in floor model. It is also worth mentioning that the weight based nature of the methadone protocol may contribute to our findings rather than the drug itself. Infants in the morphine treatment group had a trend toward increased requirement for feeding interventions, though this was not statistically significant. This accounted for some of the wider variance seen in the length of stay for the morphine treatment group. While our sample size was too small to detect any significant differences between groups, it is possible the heightened withdrawal experienced by infants in the morphine group, perhaps secondary to lower morphine equivalents, resulted in poorer feeding. This issue is likely protocol specific and does not preclude a different morphine weaning model that would prevent heightened withdrawal. Many institutions use weight based morphine protocols whereas our morphine protocol was score based in the model of past trials [26].

Baseline characteristics of polysubstance use in pregnancy in the study were different but additional stratified analysis was not possible due to small sample size. There was a trend toward more polysubstance use in the morphine group, which likely impacted length of stay and treatment, as polysubstance use is known to complicate opioid withdrawal [1, 3, 29]. Of note, the rate of tobacco use was lower than previously described studies [29], likely due to regional and temporal variation, with less tobacco used in the West and in the past 5 years during pregnancy [30]. Future studies exploring the impact of polysubstance exposure in utero and the impact on neonatal opioid withdrawal are warranted.

There was a statistically significant difference in morphine equivalents received with infants treated with methadone receiving three times the morphine equivalents of opioid medication due to the weight based loading/taper methadone protocol. Infants in the methadone treatment group also experienced 3 adverse events of over sedation requiring transfer to higher level of care (NICU). These two factors are likely related and may represent an important area of modification of the existing loading/taper weight based methadone protocol currently in use at our institution. The ideal amount of opioid may be a balance between these two extremes, or increasing the initial morphine dose to 0.05 mg/kg per dose as is used in several ESC protocols may also potentially allow for improved treatment of NOWS [31, 32].

In our study, both of our treatment groups had a lower pharmacological treatment rate (46%) than many previously published studies. This rate was also lower than our own institutional historical average of approximately 65% from previous internal analyses. These changes are likely a result of an increase in nonpharmacological measures include rooming in, skin to skin care, and encouragement of breastfeeding among our opioid exposed infants. Given the lower pharmacological treatment rate, a larger multi-center trial would be required to achieve a sample size to detect small differences in length of stay between treatment protocols.

Conclusions from our study also are also limited by the change in inclusion criteria to include babies with a < 24 h NICU stay after several months. This decision was made to increase possible participation after it was noted that several infants were missed for enrollment due to brief post-birth transitional observation that was not thought to relate to or impact opioid withdrawal. The criteria for admission to a routine newborn floor may differ between institutions, especially in the late preterm period, and thus is an important consideration in future study design. In addition, we chose to end our study without reaching stopping criteria, primarily due to the pilot study nature of our protocol and desire from a clinical standpoint to pursue more modern assessment and treatment for neonatal opioid withdrawal.

After completion of our study, researchers developed a Core Outcome Set for Neonatal Opioid Withdrawal Syndrome to guide future research efforts [33]. Our investigation included several of these core outcomes including need for pharmacologic treatment, total dose of opioid treatment, duration of treatment, feeding difficulties, parent-infant bonding (rooming-in), length of stay, breastmilk at discharge, weight gain at discharge. Our study did not include measurements of consolability, time to adequate symptom control, readmission rates for withdrawal, or developmental outcomes. Future research should include those measures.

## Conclusions

Both methadone and morphine are reasonable treatments for infants with NOWS who require pharmacologic treatment. The ideal treatment protocol for NOWS, including variables such as weight based versus score based, escalation versus load and taper, and the specific opioid medication used, are still to be determined and warrant further research. Regardless of the pharmacologic protocol used, non-pharmacologic treatments including rooming in, breastfeeding, and environmental changes should be maximized to limit the need for opioid medication and prolonged hospital stay.

## Supplementary Information

**Additional file 1.** Methadone and Morphine treatment protocols.

## Data Availability

The datasets used and/or analyzed during the current study are available from the corresponding author on reasonable request.

## Abbreviations

NOWS: Neonatal Opioid Withdrawal Syndrome
LOS: Length of Stay
LOT: Length of Treatment
NICU: Neonatal Intensive Care Unit
