# Supplementary material for: Morphine versus methadone for neonatal opioid withdrawal syndrome: a randomized controlled pilot study
Source: BMC Pediatr. 2022 Jun 15;22:345. doi: 10.1186/s12887-022-03401-3 (PMC9202148; doi:10.1186/s12887-022-03401-3)
Supplement: Supplementary file 1 — Additional file 1. Methadone and Morphine treatment protocols. [file 12887_2022_3401_MOESM1_ESM.pdf]

UNM HOSPITALS

Applies To: UNM Hospitals<br>Responsible Department: ICN<br>Revised: 1/2015	
Title: **Neonatal Abstinence Syndrome Scoring Tool**	**Guideline**
**Patient Age Group:** ( ) N/A ( ) All Ages (X) Newborns	( ) Pediatric ( ) Adult

DESCRIPTION/OVERVIEW

This guideline describes the use of the Finnegan Neonatal Abstinence Syndrome Scoring Tool for infants undergoing opioid withdrawal. This tool will be used for known drug exposed infants or infants that begin to exhibit central nervous system irritability and gastrointestinal dysfunction in the first postnatal week.

REFERENCES

- D'Apolito, K. (2010). *Assessing signs & symptoms of neonatal abstinence using the Finnegan scoring tool.* An inter-observer reliability program. Nashville, Tennessee: Neo Advances.
- Finnegan, L. P., Kron, R. E., Connaughton, J. F., & Emich, J. P. (1975). Assessment and treatment of abstinence in the infant of the drug-dependent mother. *International Journal of Clinical Pharmacology and Biopharmacology, 12*(1-2), 19-32.
- Lucas, K. & Knobel, R. (2012). Implementing practice guidelines and education to improve care of infants with neonatal abstinence syndrome. *Advances in Neonatal Care, 12*(1), 40-45. doi: 10.1097/ANC.0b013e318241bd73

AREAS OF RESPONSIBILITY

Licensed nurses working with infants in Mother Baby Unit (MBU), Newborn Nursery (NBN), Women's Special Care Unit (WSC), Carrie Tingley Hospital Inpatient Unit (CTH), Pediatric Specialty Care (PSC), Intermediate Care Nursery 3 and 4 (ICN3 and ICN4), Newborn Intensive Care Unit (NBICU), and General Pediatric Unit (GPU) where this scoring tool is used will follow this guideline.

GUIDELINE PROCEDURES

1. The total abstinence score is determined by adding the score assigned to each symptom observed through the scoring interval (e.g. any sign or symptom present during the 2-4 hour interval).
2. Start scoring when the infant is 2 hours old. Scoring interval will then continue every 2-4 hours.
3. Two licensed nurses must independently score the baby the first time for every shift and with every change of caregiver. Inter-rater reliability should be 90-100% (i.e. less than or equal to two different scoring values). If less, a third licensed nurse or provider needs to score the baby.
4. Scores include symptoms noted at any time during the 2-4 hour interval.
5. If the infant's total score is 8 or greater, score every 2 hours (if awake) or 3 hours (if asleep at the 2 hour mark) until a total score of 7 or less is obtained with every 2-3 hour scoring for a 24 hour period. After that, resume scoring every 3-4 hours.
6. If the infant does not require pharmacologic treatment by 96 hours of age, scoring may be discontinued. In general, after 96 hours without pharmacological treatment, further inpatient observation for withdrawal symptoms is not needed.
7. Non-pharmacologic (supportive measures) to be used for scores less than 8.
8. It is recommended that pharmacologic treatment be initiated if:
    a. The infant receives a total score of 8 or greater on 3 consecutive scorings or the average of any three consecutive scores is 8 or greater
    b. The total score is 12 or greater for 2 consecutive scoring intervals
    c. The average of any 2 consecutive scores is 12 or greater

SUMMARY OF CHANGES

This document combines the 2 previously used documents, "Neonatal Abstinence Scoring Tool" used by NBICU and "Neonatal Abstinence Scoring (NAS) non-ICU" used by MBU, WSC, NBN, CTH, ICN3, ICN4, PSC, and GPU.
Replaces "Neonatal Abstinence Scoring (NAS Tool)", last revision 8/1/2013.

RESOURCES/TRAINING

Resource/Dept	Contact Information

DOCUMENT APPROVAL & TRACKING

Item	Contact	Date	Approval
Owner	Intermediate Care Nurseries		
Consultant(s)	Nicole Urrea MD, Co-Medical Director ICN3; Lawrence Leeman MD, Co-Medical Director MBU and ICN3; Jennifer Rael, MD, Medical Director NBICU/ICN4; Janet Renn-Nelson, MSN, RN, RNC-NIC, CCRN, NBICU/ICN UBE; Rebecca Brown, RN, MBU/NBN UBE; Loryn Udell, BSN, RN, RNC-NIC, NBICU/ICN UBE		
Committee(s)	Clinical Operations PP&G Committee, Nursing PP&G Subcommittee		Y
Nursing Officer	Sheena Ferguson, Chief Nursing Officer		Y
Medical Director	Nicole Urrea, MD, Co-Medical Director ICN3		Y
Official Approver	Sheena Ferguson, MSN, CNS, CCRNr, CNO		Y
Official Signature		Date: 2/11/2015	
Effective Date		2/11/2015	
Origination Date		8/1993	
Issue Date	Clinical Operations Policy Coordinator	2/23/2015	ar

ATTACHMENTS

Attachment A: Prenatal Exposure to Opiates with Elevated NAS Scores Treatment Algorithm (for Medical Providers)
Attachment B: Pharmacologic Treatment Guidelines (for Medical Providers)
Attachment C: Methadone Withdrawal Guidelines (for Medical Providers)
Attachment D: Short Acting Oral Morphine for Neonatal Abstinence Syndrome (for Medical Providers)
Attachment E: IV Morphine for Neonatal Abstinence Syndrome (for Medical Providers)

Attachment A: Prenatal Exposure to Opiates with Elevated NAS Scores Treatment Algorithm (for Medical Providers)

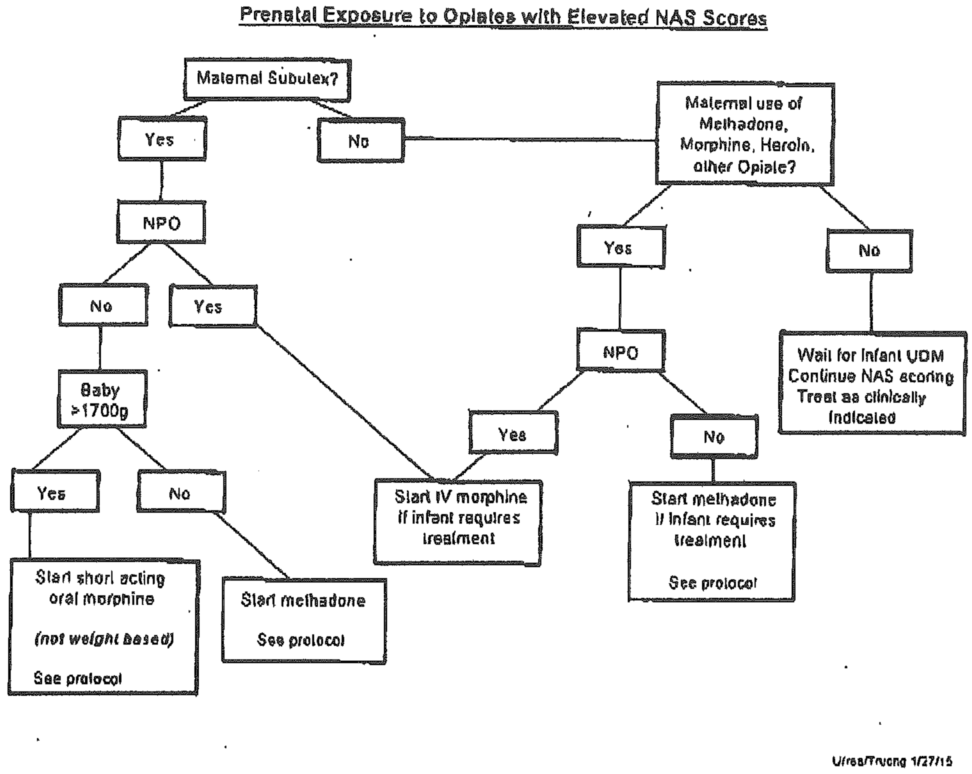

Attachment B: Pharmacologic Treatment Guidelines (for Medical Providers)

1. Methadone (except when NPO) is given for withdrawing infants when the mother has been treated with methadone, prescription narcotics (e.g. oxycodone or hydrocodone) or mom has been using heroin.
2. Morphine is given for withdrawing infants when the mother has been treated with buprenorphine (Subutex) or when infant is NPO.
3. Teach the family that the baby will have a minimum length of stay of 96 hours for observation for neonatal abstinence syndrome regardless of the type of opiate or treatment. If pharmacologic treatment for NAS is started, the length of stay will be longer.

Methadone Treatment

1. The dosing is weight based.
2. If the infant's total score is 8 or greater, score every 2 hours until a total score of 7 or less is obtained (with every 2 hour scoring) for a 24 hour period. At this time, resume scoring every 3-4 hours. Remember that scored symptoms are anything noted in the scoring interval and that baby does not need to be awake to be scored.
3. If the infant does not require pharmacologic treatment by 96 hours of age, scoring may be discontinued. In general, after 96 hours without pharmacological treatment, further inpatient observation is not needed.
4. Non-pharmacologic (supportive measures) to be used for scores less than 8.
5. It is recommended that pharmacologic treatment be initiated if:
   a. The infant receives a score of 8 or greater on 3 consecutive scorings or the average of any three consecutive scores is 8 or greater
   b. The score is 12 or greater for 2 consecutive scoring intervals
   c. The average of any 2 consecutive scores is 12 or greater

Morphine Treatment

1. The dosing is not weight based. The dosing is based on the infant's Finnegan withdrawal score.
2. Score the infant every 3-4 hours before feeds. If the infant scores 9-12, re-score after feeding or within the hour. Reassessment occurs immediately after feeds or within 1 hour. If the re-score is greater than 9, the provider will start treatment based on the highest score. If the re-score is 0-8, treatment should not be initiated.
3. If the initial score is 13 or greater, notify providers for orders to start treatment immediately without reassessment.
4. Doses are given every 3-4 hours with feeds. It is appropriate to wait up to 4 hours if the infant is sleeping. Do not exceed 4 hours between doses.

Vital Signs

1. For infants not on continuous monitoring, oxygen saturation and respiratory rate must be assessed between 30-60 minutes after the first two doses of methadone or morphine and between 30-60 minutes after each dose increase. Oxygen saturation and respiratory rate should be monitored every shift and as needed.

Attachment C: Methadone Withdrawal Guideline (for Medical Providers)

How Do I Order NAS Scoring?

Write the following order:

1. Initiate NAS scoring if the infant begins demonstrating any symptoms of mild withdrawal.

How Do I Determine If An Infant Needs Methadone?

1. The infant receives a score of 8 or greater on 3 consecutive scorings or the average of any three consecutive scores is 8 or greater
2. The score is 12 or greater for 2 consecutive scoring intervals
3. The average of any 2 consecutive scores is 12 or greater

Instructions for Dosing Methadone:

1. Determine infant's weight in kg.
2. Determine the total dose, starting with 0.7mg/kg/24 hours.
3. Divide the total dose by the desired number of doses.

Instructions for Weaning Methadone:

1. Begin the weaning steps (i.e. Step 2) when infants Finnegan scores are less than or equal to 8 for 48 hours on the Step 1 dose.
2. If scores are elevated, options are a single rescue dose or returning to the previous step.
3. Remember that every infant has a unique metabolism and may require adjustments to this scale. For example, some infants may need to be weaned more slowly if they are still scoring on the withdrawal scale.
4. Observe the infant for 48 hours after the last dose.

STEP	TOTAL DOSE	FREQUENCY
1	0.7mg/kg/24 hours	Q 4 hours
2	½ of Step 1 dose	Q 4 hours
3	Step 2 dose	Q 6 hours
4	Step 2 dose	Q 8 hours
5	Step 2 dose	Q 12 hours
6	½ of Step 5 dose	Q 12 hours
7	Day 6 dose	Q day or every other day

Attachment D:
Short Acting Oral Morphine for Neonatal Abstinence Syndrome for Bupenorphine Exposed Infants (for Medical Providers)

Morphine (0.04mg/0.2ml)
Dose given every 3-4 hours with feeds, do not exceed 4 hours between doses
For infants greater than 34 weeks and greater than1700 grams exposed to Subutex

Score	Dose for Initiation (**NOT WEIGHT BASED**)
0-8	None
9-12	0.04 mg/dose
13-16	0.08 mg/dose
17-20	0.12 mg/dose
21-24	0.16 mg/dose
≥25	0.2 mg/dose

Score Morphine Initiation:
- If neonate scores 9-12, re-score after feeding or within the hour and if the re-score is greater than 9, start treatment based on the highest score. If the re-score is 0-8, do not initiate treatment.
- If initial score is 13 or greater, start treatment immediately without reassessment.

Morphine Maintenance/Escalation:
- Maintain dose if score 0-8.
- Increase dose by 0.02 mg/dose if score is 9-12 (re-score before dosing).
- Increase dose by 0.04 mg/dose if score 13-16.
- Increase dose by 0.06 mg/dose if score 17-20.

Weaning Instructions:
- Maintain on dose for 48 hours before starting wean.
- Wean by 0.02 mg/dose of morphine every day for a score of 0-8.
- Defer wean for a score of greater than 9.

Re-escalation (after initial wean):
- If neonate scores 9-12, re-score as described for initiation. If the re-score is 9-12, increase morphine by 0.01 mg/dose every 3-4 hours.
- If 2 consecutive scores 13-16, increase morphine by 0.02 mg/dose every 3-4 hours.
- If 2 consecutive scores 17-20, increase morphine by 0.04 mg/dose every 3-4 hours.

Timing of scoring: Hospitalized infants scored every 3-4 hours before feeds.
Reassessment: Occurs immediately after feeds or within 1 hour.

Note: If an infant is on a higher than normal dose (for example, patient had previously been on IV morphine), may consider weaning by 10-20% of daily dose.

Attachment E: IV Morphine for Neonatal Abstinence Syndrome (for Medical Providers)

Morphine concentration = 1mg/ml
Dose given every 3-4 hours with feeds or cares (if NPO); do not exceed 4 hours between doses.
IV route is for infants who cannot take oral medications, regardless of gestational age.

Score	Dose for Initiation (**NOT WEIGHT BASED**)
0-8	None
9-12	0.015 mg/dose
13-16	0.03 mg/dose
17-20	0.045 mg/dose
21-24	0.06 mg/dose
≥25	0.075 mg/dose

Score Morphine Initiation:
- If neonate scores 9-12, re-score after feeding or within the hour and if the re-score is greater than 9, start treatment based on the highest score. If the re-score is 0-8, do not initiate treatment.
- If initial score is 13 or greater, start treatment immediately without reassessment.

Morphine Maintenance/Escalation:
- Maintain dose if score 0-8.
- Increase dose by 0.0075 mg/dose if score is 9-12.
- Increase dose by 0.015 mg/dose if score 13-16.
- Increase dose by 0.025 mg/dose if score 17-20.

Weaning Instructions:
- Maintain on dose for 48 hours before starting wean with scores less than 8.
- Wean by 0.002-0.004 mg/dose of morphine every day for a score of 0-8.
- Defer wean for a score of greater than 9.

Re-escalation (after initial wean):
- If neonate scores 9-12, re-score as described for initiation. If the re-score is 9-12, increase morphine by 0.00375 mg/dose every 3-4 hours.
- If 2 consecutive scores 13-16, increase morphine by 0.0075 mg/dose every 3-4 hours.
- If 2 consecutive scores 17-20, increase morphine by 0.0125 mg/dose every 3-4 hours.

Converting IV to Oral morphine:
- Consider a 1:2 conversion when converting IV morphine to oral morphine (1 mg of IV morphine is approximately equivalent to 2 mg of PO morphine). May consider consulting with NICU pharmacist when converting to oral morphine. For example: if receiving 0.06 mg/dose of IV morphine, the oral morphine dose would be 0.12 mg/dose.

Timing of scoring: Hospitalized infants scored every 3-4 hours before feeds or care times.
Reassessment: Occurs immediately after feeds (or care times) or within 1 hour.

Note: If an infant is on a higher than normal dose, may consider weaning by 10-20% of daily dose.

Clonidine as adjunct to methadone for severe neonatal abstinence: Pilot Protocol (Nov-2013)

Background: Opiates are the drug of choice for the pharmacological treatment of neonatal abstinence syndrome (NAS) with common options including methadone, morphine and tincture of opium. Phenobarbital and clonidine have been used as adjunct agents in infants who have continued high NAS scores despite treatment with opiates. We are using clonidine as an adjunct for neonates who either have

1) Inadequate treatment of their NAS despite using methadone 0.7mg/kg /day divided q 4 hr starting dose for at least 2 doses. Increasing the dose of methadone is another option for these infants. Either option requires a monitored setting in ICN3 or NICU
2) Inability to initiate weaning from oral methadone as evidenced by 5 or more days at the same q 4 or q 6 hour dosing interval

Protocol

1) Infant needs to be on cardiopulmonary monitoring in ICN-3 or NICU. Check blood pressures 30-60 minutes after first two doses and then q shift
2) Parents informed of need for additional medicine
3) Opiate continued at current dose when clonidine started
4) Start oral clonidine at 1 microgram/kg every 4 hours (20 mcg/cc solution)
5) Once NAS scores have decreased to an acceptable range for weaning methadone then resume usual methadone weaning protocol while keeping clonidine at starting dose and interval
6) When methadone reaches q 12 hour dosing, then start weaning clonidine while maintaining stable methadone dose
7) Decrease clonidine to q 6 hours for four doses (24 hours), then q8 hours for three doses (24 hours), then q 12 hours for two doses (24 hours) then stop. Hold wean if NAS scores worsen to >8 on two consecutive scores or the average of three in a row is >24.
8) Check BP q shift while on clonidine and for the first 48 hours after final dose of clonidine
9) Do not wean methadone when clonidine is being weaned. Once off clonidine resume usual methadone weaning protocol.
10) Infants on clonidine or within 96 hours of being weaned need to remain on monitored unit

References

Clonidine as an Adjunct Therapy to Opioids for Neonatal Abstinence Syndrome: A Randomized Controlled Trial Agthe, et al. Pediatrics 2009; 123:e849–e856

Neonatal Drug Withdrawal Mark L. Hudak, MD, Rosemarie C. Tan, MD, PhD, AAP Clinical Report Pediatrics Vol. 129 No. 2 February 1, 2012 pp. e540 -e560
